# Supplementary material for: Microsaccades transiently lateralise EEG alpha activity
Source: Prog Neurobiol. 2023 May;224:102433. doi: 10.1016/j.pneurobio.2023.102433 (PMC10074474; doi:10.1016/j.pneurobio.2023.102433)
Supplement: Supplementary file 1 — Supplementary material [file mmc1.docx]

**Supplementary Information – Figures S1-S5**

**
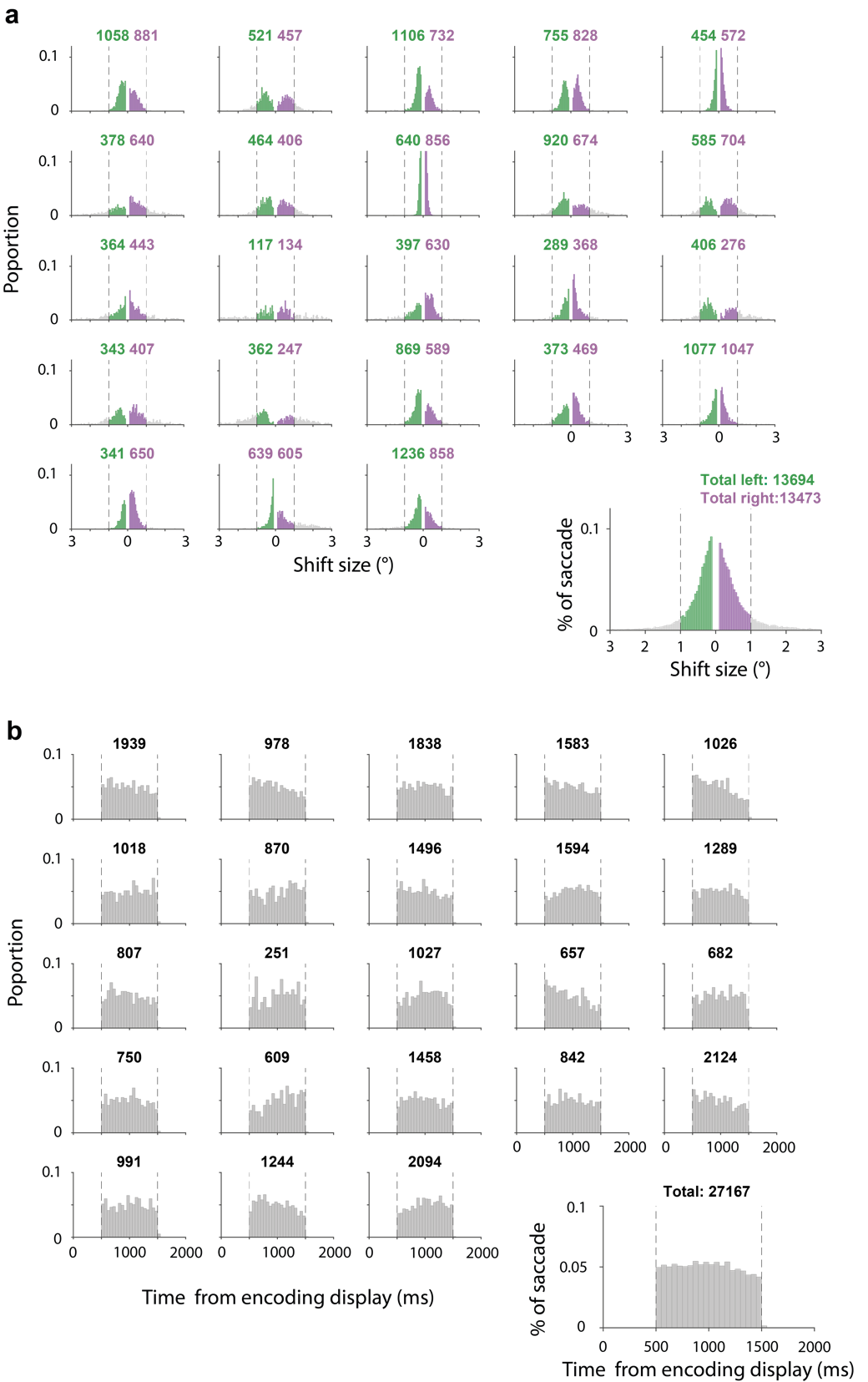
**

**Supplementary Figure 1. Spatial and temporal distributions of identified microsaccades across participants.** **a)** Direction and magnitude distributions of detected horizontal saccades during the retention delay. The dashed line represents the threshold (1-degree visual angle) under which the detected saccades were treated as ‘microsaccades’ and used in the reported analysis. **b)** The temporal distribution of the identified usable microsaccades across the delay period. In both panels, each plot represents an individual participant, while the right bottom plot represents the distribution of detected microsaccades aggregated across all participants.


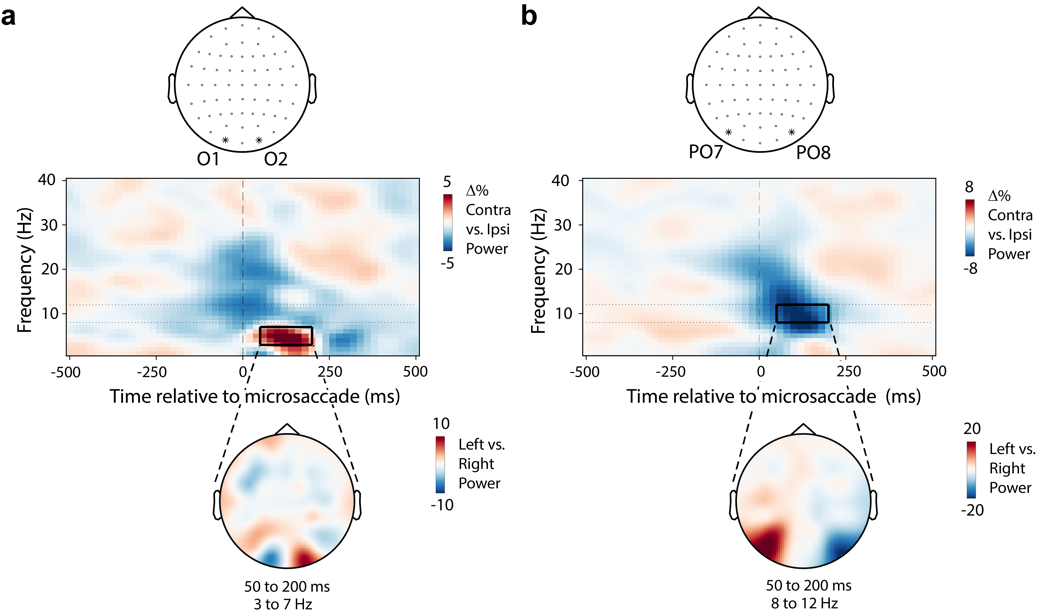


**Supplementary Figure 2. Microsaccade-locked EEG responses in 3-7 Hz and 8-12 Hz have distinct topographies and opposite directions of modulation.** **a)** Analyses like in **Main Figure 1**, but instead of using electrodes PO7/8, we used electrodes O1/O2. **b)** equivalent results using electrodes PO7/8 instead for reference (same data as in **Main Figure 1**).

**
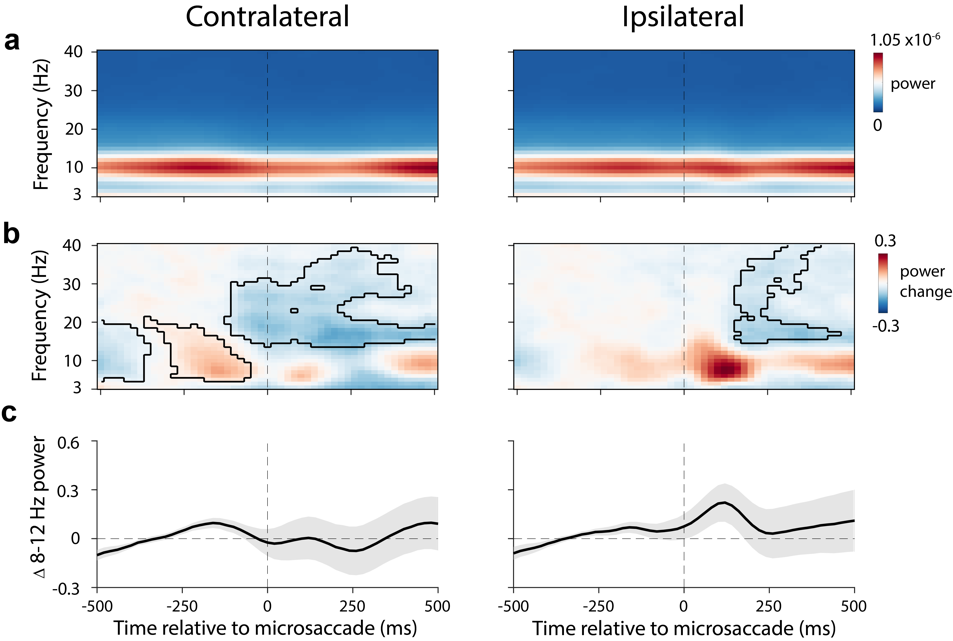
**

**Supplementary Figure 3. Similar to main Figure 3, using return microsaccades instead of start microsaccades.** Note how the observed time-frequency clusters should be interpreted with caution because, for return microsaccades, the pre-microsaccade baseline is likely compromised by neural modulations in response to preceding start microsaccades. Accordingly, the observed clusters may at least in part reflect modulations due to start microsaccades that preceded return microsaccades.

**
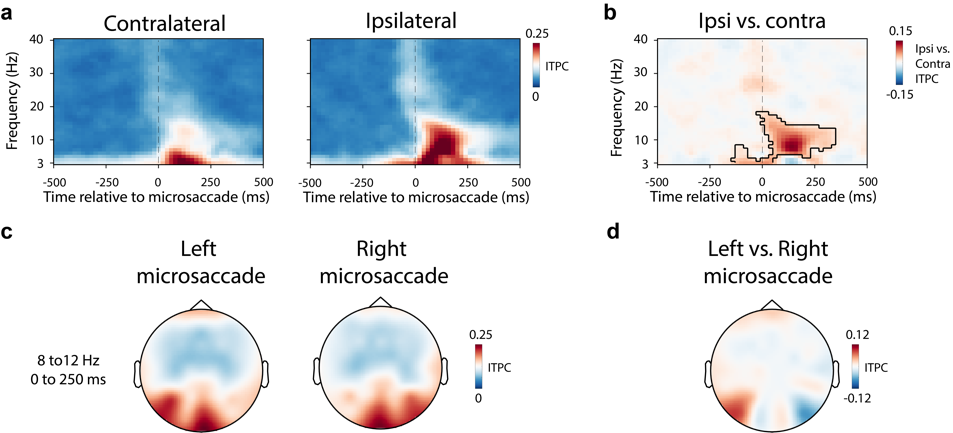
**

**Supplementary Figure 4. Replication of main Figure 4, using return microsaccades instead of start microsaccades.**

**
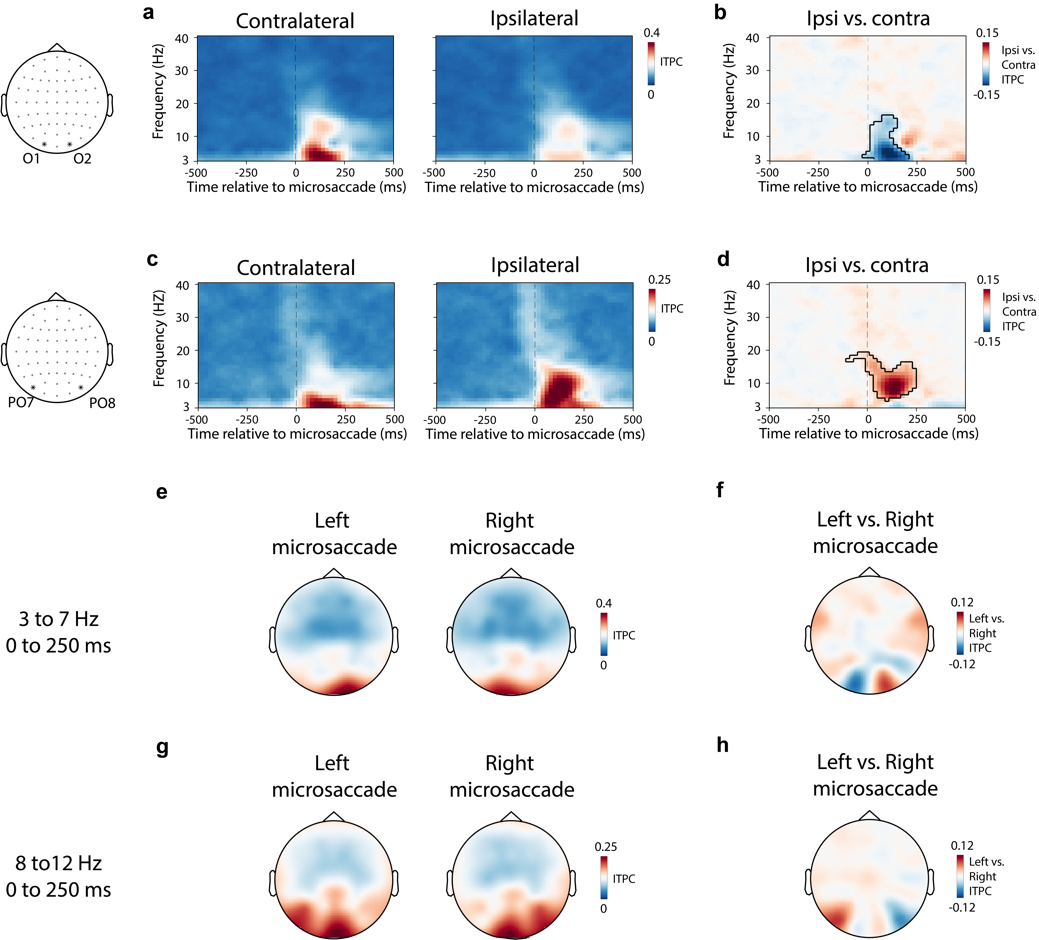
**

**Supplementary Figure 5. Inter-trial phase-coherence as a function of electrodes (O1/2 and PO7/8).** Conventions as in **Main Figure 4**, separately for electrodes O1/2 (a-b) or PO7/8 (c-d) and for 3-7 Hz (e-f) and 8-12 Hz (g-h).
